# Highly porous core–shell chitosan beads with superb immobilization efficiency for *Lactobacillus reuteri* 121 inulosucrase and production of inulin-type fructooligosaccharides†

**DOI:** 10.1039/c8ra02241k

**Published:** 2018-05-09

**Authors:** Thanapon Charoenwongpaiboon, Karan Wangpaiboon, Rath Pichyangkura, Manchumas Hengsakul Prousoontorn

**Affiliations:** Department of Biochemistry, Faculty of Science, Chulalongkorn University Payathai Road Bangkok 10330 Thailand manchumas.h@chula.ac.th

## Abstract

With the aim to overcome the limitations of hydrogel chitosan beads (HGBs), various types of chitosan, core–shell chitosan beads (CSBs), and dried chitosan beads (DBs) were synthesized. Physical and chemical properties were compared with those of HGBs. CSBs were proved to be an effective support because they displayed higher stability and capacity over the HGBs, and thus, were selected for enzyme immobilization. Recombinant inulosucrase (INU) from *Lactobacillus reuteri* 121 was immobilized on CSBs using glutaraldehyde as a cross-linker. Immobilized biocatalysts (INU-CSBs) were then used for the synthesis of inulin-type fructooligosaccharide (IFOS). Biochemical characterization revealed that the optimum pH of both INU-CSBs and free enzyme was unaltered at 5.5 whereas the optimum temperature of INU-CSBs shifted from 50 °C to 60 °C. Moreover, pH stability and thermostability of INU-CSBs significantly improved. For batch synthesis of IFOS, INU-CSBs retained approximately 45% of their initial catalytic activity after being reused for 12 cycles. IFOS was also continuously synthesized in a fixed-bed bioreactor for a reaction duration of at least 30 h. The high efficiency of INU-CSBs makes them very attractive for industrial applications.

## Abstract 2

Inulosucrase immobilized on chitosan bead in core–shell format has proved to be an attractive biocatalyst for the synthesis of inulin-type fructooligosaccharides.

## Graphical abstract

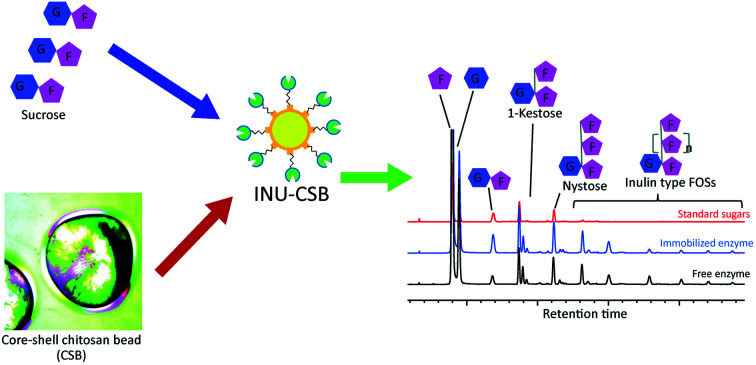

## Introduction

Inulin-type fructooligosaccharides (IFOSs) are oligomers of fructose in which fructose residues are covalently linked *via* a β(2→1) glycosidic linkage. IFOSs are well known as good prebiotics because they are indigestible by the human gastrointestinal tract^1^ and thus provide only a small amount of energy.^2^ Normally, IFOSs are produced *via* enzymatic activity of β-fructofuranosidase (E.C. 3.2.1.26) derived from various fungal species such as *Aspergillus niger*,^3,4^*Aspergillus japonicas*,^5–7^*Aspergillus aculeatus*,^8,9^*Aspergillus awamori*,^10^*Aspergillus kawachii*,^11^ and *Aspergillus oryzae*.^12^ Nonetheless, the use of fungal enzymes had been shown to have some limitations because they prefer hydrolysis to transfructosylation, and most of the fungal enzymes specifically synthesize only a short chain of IFOS with a degree of polymerization (DP) in the range of 2–4.

Inulosucrase (E.C. 2.4.1.9) is a bacterial fructosyltransferase that can synthesize IFOS from sucrose. Inulosucrase has been identified in only a few bacterial species such as *Leuconostoc citreum*,^13^*Lactobacillus reuteri*,^14^*Lactobacillus johnsonii*,^15^*Lactobacillus gasseri*,^16–18^ and *Streptomyces viridochromogenes*.^19^ Structural analysis shows that bacterial inulosucrases have low structural similarity when compared to fungal β-fructofuranosidase. In contrast, they are closely related to levansucrase (E.C. 2.4.1.10), another type of fructosyltransferase, which synthesizes mainly β(2→6) levan-type fructooligosaccharides. Production of IFOS using inulosucrase has an advantage (over fungal β-fructofuranosidase) because it provides high-molecular-weight inulin and longer chains of IFOS. In addition, inulosucrase possesses high transglycosylation activity and thus is suitable for an acceptor reaction. Nevertheless, the stability of bacterial fructosyltransferase is lower than that of the fungal enzyme.

To increase the stability of a biocatalyst, the enzyme can be prepared in an immobilized form. Glutaraldehyde-mediated immobilization is a method where an enzyme and support, in some cases an enzyme and enzyme, are covalently linked by means of glutaraldehyde as a cross-linker.^20^ The formation of a covalent bond is irreversible. Therefore, the enzyme cannot be released even though the reaction conditions are changed. Chitosan-based materials have usually been employed as support because they are eco-friendly, low-cost, nontoxic and biodegradable. Furthermore, the presence of readily available amino groups in chitosan makes it readily reactive with the aldehyde group of glutaraldehyde. As a result, glutaraldehyde-activated chitosan beads can be prepared. Chitosan beads are generally prepared in a hydrogel form by a neutralization method.^21^ This method is easy to use but the volumetric activity of the biocatalyst is quite low. Moreover, the structure of hydrogel beads is known to be jellylike and therefore can be easily distorted when the beads are facing a mechanical force or pressure. To achieve higher efficiency of supporting carriers, chitosan can be combined into a composite with various inorganic materials including silica,^22–24^ graphene oxide,^25,26^ and various metal nanoparticles.^27,28^ Although the inorganic/chitosan composites have better properties than nonderivatized chitosan, the preparation of composite materials is usually complicated and incurs a high cost, which is not suitable when they are applied on an industrial scale.

In recent years, there have been many studies on the synthesis of fructooligosaccharides using immobilized bacterial levansucrase and fungal β-fructofuranosidase.^29^ Nonetheless, to the best of our knowledge, there have been no reports on the immobilization of bacterial inulosucrase. Due to the structural difference between inulosucrase and β-fructofuranosidase, it is very interesting to study the production of IFOS by means of immobilized inulosucrase. In this work, various chitosan beads, namely porous core–shell chitosan beads (CSBs) and dried chitosan beads (DBs), were prepared to overcome some limitations of the jellylike form. Physical properties of the synthesized beads including surface morphology, porosity, and protein-binding capacity were studied and compared with those of normal hydrogel chitosan beads (HGBs). After that, the best chitosan beads were applied as a carrier for enzyme immobilization. Inulosucrase from *Lactobacillus reuteri* 121 (a single subunit enzyme with the MW of ∼87 kDa)^14^ was bound onto the chosen support through a covalent linkage *via* glutaraldehyde. The effects of immobilization conditions such as pH, glutaraldehyde, and enzyme concentration were investigated. Biochemical properties of the immobilized enzyme were explored and compared to those of the free enzyme. Finally, the performance of the immobilized inulosucrase was further evaluated with regard to the production of IFOS in both batch and continuous processes.

## Experimental

### Materials

Chitosan polymer (MW > 600 000, degree of deacetylation > 80%) was obtained from Oilzac Technologies Co., Ltd (Thailand). Standards of sugar 1-kestose and nystose were purchased from Sigma-Aldrich.

### Enzyme expression and purification

The gene of inulosucrase from *Lactobacillus reuteri* 121 (inu; GenBank accession number AF459437) was synthesized by Genscript. The synthetic gene was subcloned into pET21-b *via* NdeI and XhoI sites. The recombinant vector (pETIns) was transformed into *Escherichia coli* BL21 (DE3). Plasmid-carrying *E. coli* strains were grown at 30 °C in the Luria-Bertani medium, supplemented with 100 μg mL^−1^ ampicillin, 10 mM CaCl_2_, and 0.1 mM IPTG for enzyme induction. After 18 h, the cells were harvested using centrifugation (5000 × *g*) at 4 °C for 10 min. The cell pellet was resuspended in 50 mM sodium citrate buffer pH 5.0 and then sonicated. Cell debris were removed by centrifugation (10 000 × *g*, 4 °C, 15 min) to obtain the crude extract of the enzyme.

Inulosucrase was partially purified by anion exchange chromatography. The crude extract was loaded onto DEAE (Toyopearl DEAE-650M) resin that was pre-equilibrated with 25 mM potassium phosphate buffer pH 7.0 at 4 °C. The protein was eluted with the same buffer containing 50 mM NaCl. The obtained enzyme was further used for immobilization. The total protein concentration was measured by Bradford's assay.

### Preparation of HGBs, DBs, and CSBs

**Fig. 1:**
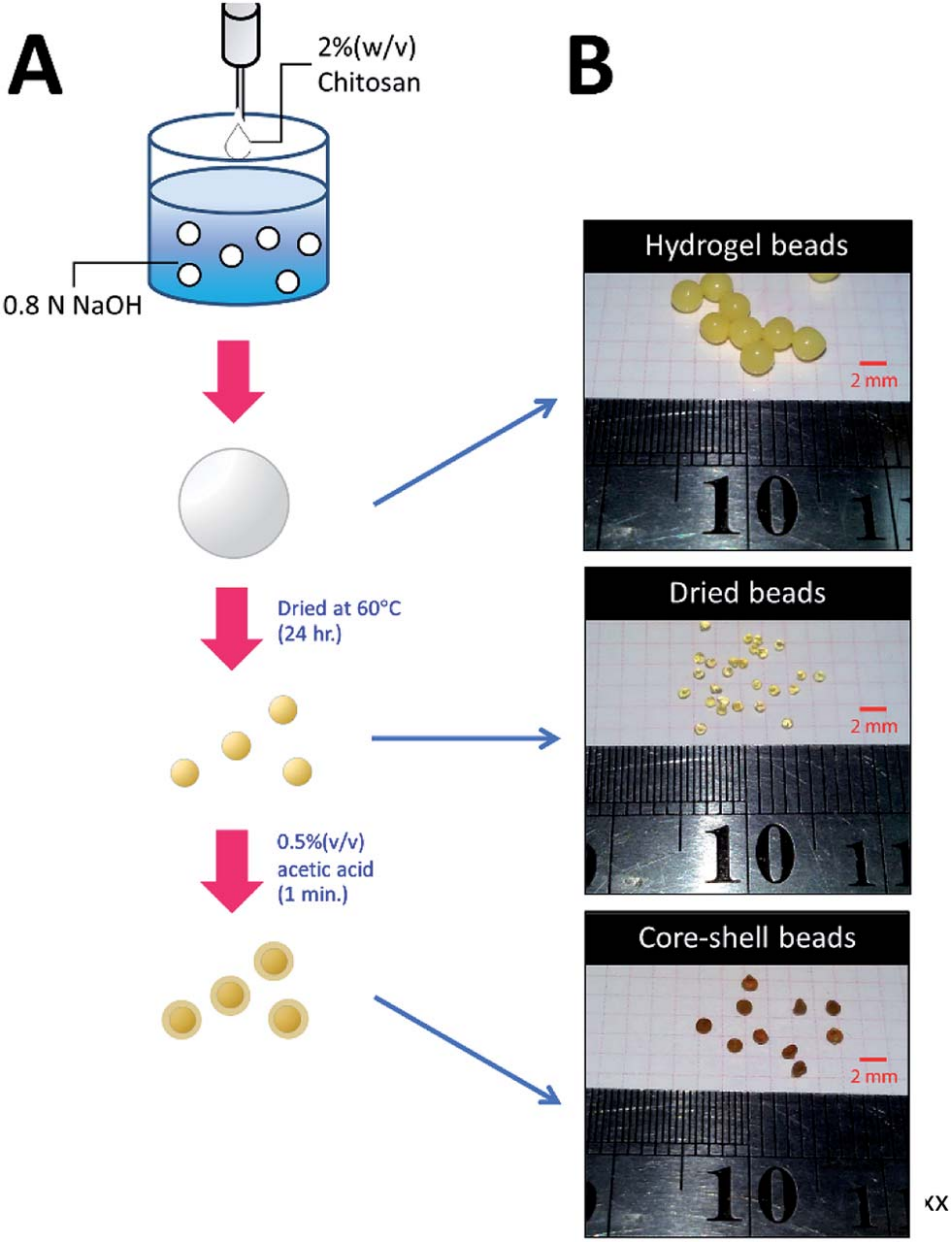
Preparation of HGBs, DBs, and CSBs. (A) A schematic diagram showing two-step processes for preparation of DBs and CSBs. (B) Photos of glutaraldehyde-cross-linked HGBs, DBs, and CSBs.

The methodology for preparing three types of chitosan beads is summarized in Fig. 1. For HGB preparation, 20 g of chitosan was dissolved in 1 L of a mixed acid solution containing 2% (w/v) of acetic acid, 1% (w/v) of lactic acid, and 1% (w/v) of citric acid. The resulting chitosan solution was introduced dropwise into 0.8 N NaOH by a peristaltic pump. The resultant HGBs were washed with deionized water until pH became neutral. DBs were prepared by drying the HGBs obtained above at 60 °C for 24 h. For CSB preparation, DBs were soaked in a 0.5% (w/v) acetic acid solution for 60 s and then neutralized by the addition of an equal molar amount of NaOH.

### Characterization of chitosan beads

To study the support capacity, an experiment on adsorption of a protein onto the chitosan beads was carried out. BSA served as a model protein. Approximately 0.1 g of DBs, HGBs or CSBs, that was preactivated with 1% (w/v) glutaraldehyde was incubated with a solution with a known concentration of BSA (C0) at 4 °C for 24 h. The equilibrium concentration of BSA (Ce) was then determined on a spectrophotometer at 280 nm using 0–2 mg mL^−1^ BSA as external standard. The adsorbed concentration (*Q*_e_) can be calculated using the following equation: *Q*_e_ = (*C*_0_ − *C*_e_)*V*/*W*, where *V* is the volume of the BSA solution and *W* is the weight of chitosan beads (g). The Langmuir equation, *Q*_e_ = KL*C*_e_/(1 + αL*C*_e_), served for estimation of monolayer saturation capacity (KL/αL), where KL and αL are the isotherm parameters. Furthermore, the morphology of DBs and CSBs was also monitored under a light microscope (Leica M165 FC). All the beads were dried by critical point dryer (CPD) and were coated with gold particles prior to the scanning electron microscopy (SEM) analysis (Jeol JSM 6400).

### Immobilization of inulosucrase on HGBs and CSBs

To attain high efficiency of the biocatalyst, the immobilization conditions, such as pH and glutaraldehyde and enzyme concentrations, were optimized. HGBs and CSBs were activated with 0.05–8.0% (w/v) glutaraldehyde in a buffer with a pH range of 5.0–8.0 (acetate buffer pH 5.0–6.0; phosphate buffer pH 7.0–8.0) at 4 °C for 24 h. After that, the activated chitosan beads were incubated with an enzyme solution (25–800 U per g of beads; or 0.97–30.3 mg per g of beads) at 4 °C for 24 h with mild agitation. The immobilized enzymes were washed with 1 M NaCl to remove an electrostatically adsorbed enzyme and then were washed 3 times with 50 mM acetate buffer pH 5.5. The resultant immobilized enzyme was kept at 4 °C until further use. The immobilized activity and activity yield served as parameters to optimize immobilization conditions. The immobilized activity was calculated as immobilized activity (U) per gram of beads and the activity yield (%) was expressed as activity on beads (U)/[initial enzyme (U) − unbound enzyme (U)] × 100.

### Enzymatic activity assay

Inulosucrase activity was determined by a DNS assay.^30^ In brief, 10 μL of an appropriately diluted enzyme solution was added into 490 μL of a sucrose solution (250 mM of sucrose in 50 mM acetate buffer pH 5.5 with 1 mM CaCl_2_) and incubated at 50 °C for 10 min. The reaction was stopped by the addition of an equal volume of the DNS reagent and then boiled for 10 min. The amount of reducing sugar was measured on a spectrophotometer at 540 nm. Glucose at concentrations 0–10 mM served as standard solutions. One unit of inulosucrase was defined as the amount of enzyme required to release 1 μmol of reducing sugar per minute under the described conditions.

### Effects of pH and temperature on the activity of free and immobilized inulosucrase

The optimal pH for both free and immobilized inulosucrase on core–shell chitosan beads (INU-CSBs) was measured in a pH range of 3.0–8.0 at 50 °C in 50 mM citrate buffer (pH 3.0–4.5), acetate buffer (pH 4.5–6.0), or phosphate buffer (pH 6.0–8.0). The optimum temperature for INU-CSBs and free enzyme was determined by assaying enzymatic activity in 50 mM acetate buffer pH 5.5 in a temperature range of 10–70 °C. For analysis of pH stability, the residual enzymatic activity was measured by preincubating the free and immobilized enzyme at 30 °C for 3 h at various pH levels in the Britton–Robinson universal buffer (pH 3–12). The thermostability of both enzyme samples was investigated by measuring residual activity of the enzyme after incubation in 50 mM acetate buffer pH 5.5 with 40 mM CaCl_2_ at 50 °C from 0 to 12 h.

### Sugar analysis

Identification of IFOS composition was conducted by high-performance anion exchange liquid chromatography with a pulsed amperometric detector (HPAEC-PAD ICS 5000 system, Dionex) and a CarboPack PA1 column. The column was equilibrated with 150 mM NaOH and then was eluted with a linear gradient of 0–250 mM sodium acetate in 150 mM NaOH for 30 min. Glucose, fructose, 1-kestose, and nystose served as external standards. Thin-layer chromatography (TLC) analysis was performed with a solvent system of acetonitrile : water (85 : 15, v/v). The TLC plate was dried and visualized by spraying with a solution containing 27 mL of ethanol, 10 mL of conc. H_2_SO_4_, 8 mL of water, and 0.1 g of orcinol.

Quantitative analysis of IFOS was performed on an HPLC system (Prominence UFLC, Shimadzu) with an amino column (Shodex Asahipak NH2P-50 4E) and a refractive index detector (SPD-M20A, Shimadzu). Samples were eluted with an isocratic solution, acetonitrile : water (65 : 35, v/v),at a flow rate of 1 mL min^−1^. The trisaccharide 1-kestose and tetrasaccharide nystose were employed as external standards for peak quantification. Glucose was quantified with a Glucose Liquicolor Kit (Human), and total reducing sugar was quantified by the DNS assay. The molar amount of fructose was calculated from the difference between the molar amounts of reducing sugar and glucose.

### Batch production of IFOS using immobilized inulosucrase

A preliminary experiment on IFOS synthesis was carried out at 40 °C. Ten units of free and immobilized inulosucrase (INU-CSBs) was incubated with 1 mL of 200 g L^−1^ sucrose containing 50 mM acetate buffer pH 5.5 and 40 mM CaCl_2_. After 24 h, IFOS syrup was sampled to analyze the composition by HPAEC-PAD. For the analysis of operational stability of the immobilized enzyme in a batch system, INU-CSBs were added into 200 g L^−1^ sucrose in 50 mM acetate buffer pH 5.5 with 40 mM CaCl_2_ to achieve the final activity of 10 U mL^−1^. The reaction was allowed to proceed in an orbital shaker at 40 °C for 2 h and then was stopped by removal of the biocatalyst. INU-CSBs were washed 3 times with cold 50 mM acetate buffer pH 5.5 for reutilization. The immobilized-enzyme activity of each production batch was measured by the method described above. The IFOS content was determined by HPLC. The IFOS yield was calculated as the percentage ratio of total IFOS (g L^−1^) to initial sucrose content (g L^−1^).

### IFOS synthesis in a continuous fixed-bed bioreactor with immobilized inulosucrase

Five hundred units' worth of INU-CSBs was packed into a small double-jacket column with the total volume of 7 mL. The reaction was carried out at 40 °C for 30 h. The feeding solution (200 g L^−1^ sucrose in 50 mM acetate buffer pH 5.5 containing 40 mM CaCl_2_) was loaded onto the column at a constant flow rate of 0.2 mL min^−1^. The reaction mixture was sampled at certain intervals to analyze it for sugar composition by HPLC.

## Results and discussion

### Expression and purification of inulosucrase

The inulosucrase from *Lactobacillus reuteri* 121 was successfully cloned and expressed in *E. coli* BL21 (DE3) with specific activity of ∼264 U per mg of protein. The recombinant inulosucrase was partially purified by anion exchange chromatography. The percent recovery of inulosucrase was 48% of the total activity with 3.9-fold purification (Fig. S1,† Table S1†).

### Preparation and characterization of HGBs, DBs, and CSBs

**Fig. 2:**
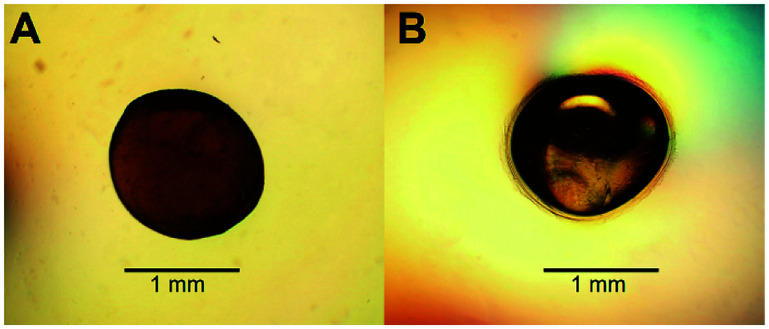
Light microscope magnification of (A) dried and (B) core–shell chitosan beads.

To increase the surface area of chitosan beads, their volume was reduced by dehydrating the interior. In the present study, HGBs were dried at 60 °C for 24 h. Light microscopy showed that the shape of the resulting DBs was nearly spherical with a diameter of approximately ∼1.3 mm (Fig. 2A, Table S2†). The reduction of bead diameter caused an increase of the surface area per volume ratio (A^2^ cm^−3^) of the beads. As presented in Fig. 1B, the size of DBs and CSBs was much smaller than that of HGBs. Furthermore, the resulting DBs were found to be more rigid than HGBs. Although this finding indicated that the compactness of the chitosan polymer after removal of adsorbed water improved the mechanical stability of the beads, the analysis of BSA adsorption revealed that protein-binding capacity of DBs was much lower than that of HGBs (Table 1, Fig. S2†). The decrease in protein-binding capacity could be explained by the fact that the reactive surface of HGBs might have collapsed after drying. To recover the protein-binding capacity of DBs, the solid surface of DBs was next modified.

**Table 1:** BSA adsorption analysis of HGBs, DBs, and CSBs

Beads	Bead density^a^ (g ml^−1^)	Protein binding capacity (mg g^−1^)	Volumetric capacity^b^ (mg ml^−1^)
HGBs	0.568 ± 0.013	5.70 ± 0.18	3.24
DBs	0.862 ± 0.010	0.182 ± 0.011	0.157
CSBs	0.878 ± 0.032	7.61 ± 0.56	6.68

a Gram of the bead packed in known volume of cylinder.

b Volumetric capacity (mg mL^−1^) = bead density (g mL^−1^) × protein binding capacity (mg g^−1^).

Reswelling of the solid beads in a dilute acetic acid solution for a while is a simple technique that can produce a highly reactive surface of solid chitosan beads. An adsorption assay using BSA as a model protein showed that the capacity of CSBs for protein binding was recovered up to 6.68 mg (mL^−1^ beads) (Table 1), which is approximately 2-fold higher than that of HGBs (3.24 mg per mL of beads) and 43-fold higher than that of DBs (0.157 mg per mL of beads). The increase in protein-binding capacity of CSBs could have resulted from the increase of their surface area after the swelling. Light microscopy indicated that there was a thin layer of a chitosan gel around a solid core (Fig. 2B). The thickness of this layer was found to be ∼0.1 mm (Table S2†). Thus, it did not significantly affect the whole size of the beads.

**Fig. 3:**
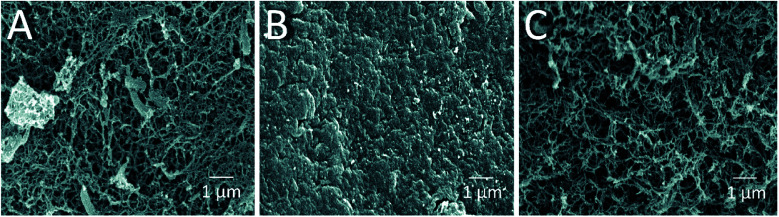
SEM micrographs of (A) HGBs, (B) DBs and (C) CSBs at magnification 10 000×.

The morphology and porous structure of the beads were then further analyzed by SEM (Fig. 3). The results showed that the external surface of HGBs and CSBs had high porosity whereas the DB surface was quite smooth. This observation indicated that reswelling process in the dilute acetic acid solution could regenerate the surface porosity but did not affect rigidity of the core. Preparation of novel core–shell beads by this simple method can overcome the limitations of the traditionally synthesized hydrogel beads. CSBs did not only ensured higher stability of organic-based support but also enabled binding of the large amount of the enzyme, and thus, CSBs were selected for enzyme immobilization.

### Immobilization of inulosucrase on HGBs and CSBs

**Fig. 4:**
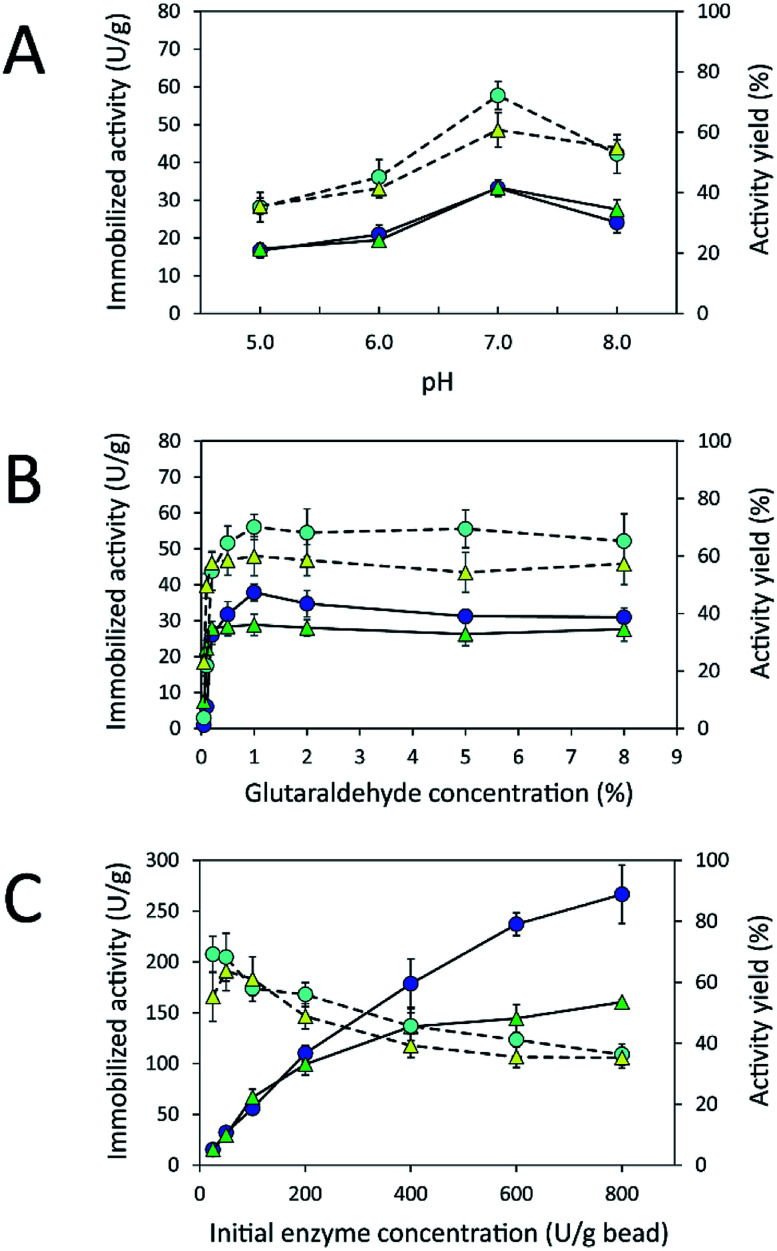
Effects of (A) pH and (B) glutaraldehyde and (C) enzyme concentrations on the immobilized activity (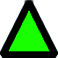, INU-HGB; 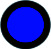, INU-CSB) and activity yield (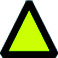, INU-HGB; 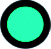, INU-CSB) of immobilized inulosucrase. The data represent means of five assays and error bars represent the standard deviation of five experiments.

Due to the high protein-binding capacity of HGBs and CSBs, they were applied as supports for the immobilization of inulosucrase. The preparation of the immobilized enzyme *via* glutaraldehyde usually involves two steps: the first is activation of amino-functionalized beads using glutaraldehyde, and the second is covalent attachment of the enzyme to the activated beads.^20,31^ The immobilization conditions such as pH and glutaraldehyde and enzyme concentrations were optimized because the structure of glutaraldehyde and thus the immobilization efficiency are largely dependent on solution conditions.^32–34^ First, the effect of pH on immobilized activity and activity yield was investigated by fixing glutaraldehyde concentration and the initial enzyme amount in units at 1% (w/v) and 50 U per g of beads, respectively (Fig. 4A). The activity assay revealed that the immobilized enzyme worked best at pH 7.0. Both INU-HGBs and INU-CSBs showed maximum immobilized activity of approximately 33.4 ± 1.5 and 33.2 ± 2.2 U g^−1^ with the activity yield of approximately 60.7% ± 5.7% and 72.1% ± 4.6%, respectively. The highest activity of immobilized enzymes at this pH might have resulted from the irreversible formation of covalent bonds between glutaraldehyde and amino groups in the pH range of 7.0–9.0.^32–34^ Moreover, higher pH is conducive to the polymeric form of glutaraldehyde rather than monomeric form and reduces steric hindrance between the enzyme and support.^32–34^

Second, the effect of glutaraldehyde concentration (0.05–8.0%, w/v) on the immobilization procedure was then investigated at pH 7.0 (Fig. 4B). The results showed that the immobilized activity and activity yield increased with an increase in glutaraldehyde concentration. Nevertheless, when glutaraldehyde concentration was higher than 1% (w/v), the immobilized activity did not increase. This finding indicated that the increase in glutaraldehyde concentration resulted in more covalent bonds per enzyme molecule and therefore may cause a conformational change of the enzyme. In addition, at a higher concentration of glutaraldehyde, there is good chance of a covalent modification close to the active site of the enzyme leading to enzyme inactivation.

**Fig. 5:**
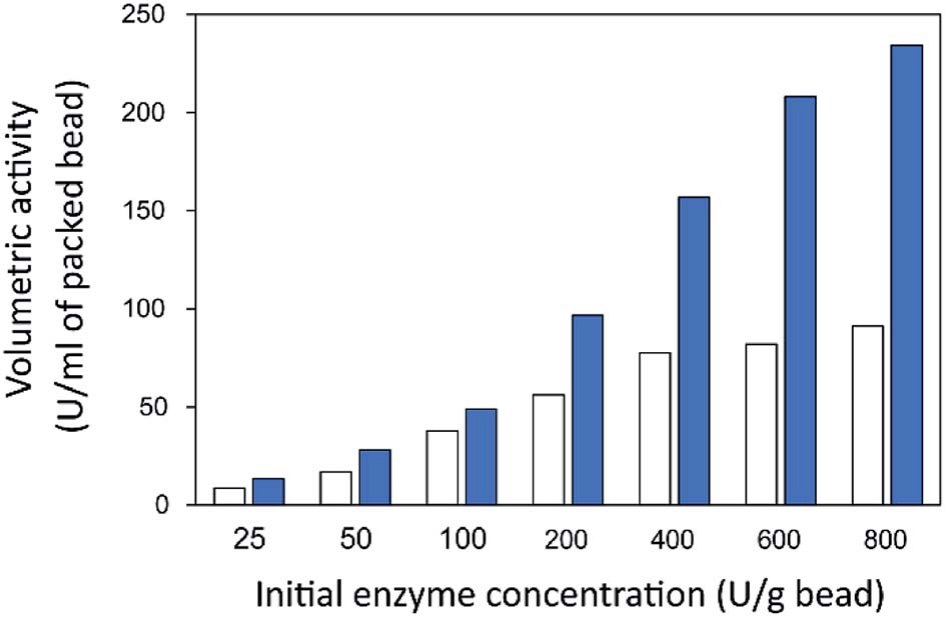
Volumetric activity of inulosucrase on immobilized HGBs (white bars) and CSBs (blue bars).

Finally, the influence of enzyme concentration on enzyme immobilization was examined by incubating activated chitosan beads (1% [w/v] glutaraldehyde), with different concentrations of the enzyme (Fig. 4C). Readers can see that when the amount of inulosucrase added per gram of beads increased from 0 to 400 U g^−1^, the activity of INU-HGBs and INU-CSBs rapidly increased. After that, the activity of both immobilized-enzyme samples reached a plateau. This result might be explained as follows: the reactive groups on the support were saturated with the enzyme. In addition, INU-CSBs showed approximately 1.5- to 2.5-fold higher volumetric activity of the packed biocatalyst as compared with INU-HGBs when the same concentration of the enzyme was added (Fig. 5). This result is consistent with the above finding that CSBs have higher protein-binding capacity than HGBs. Although the immobilized activity of both immobilized enzymes increased with enzyme concentration, their activity yield was found to decrease. The substantial enzyme loading on the support generally leads to a reduction in the activity yield owing to steric hindrance. Consequently, substrates were prevented from accessing the active site of the enzyme. This could also result from the diffusional effect of substrate and product to and from the immobilized enzyme molecules. For further analysis, an enzyme concentration of 50 U g^−1^ was chosen because it provided the highest activity yield of approximately 63.7% ± 6.5% and 68.2% ± 7.8% for INU-HGBs and INU-CSBs, respectively.

The homology modeling of the three-dimensional (3D) structure of inulosucrase revealed that the immobilization *via* a glutaraldehyde linkage is appropriate because lysine residues, which can readily react with the aldehyde group of glutaraldehyde, are located on the enzyme surface, not in the active site (Fig. S3†). This finding has been reported in the case of levansucrase from *Z. mobilis* immobilized on vinyl-sulfone activated silica.^35^ Because INU-CSBs had higher stability and activity of the immobilized enzyme than INU-HGBs did, INU-CSBs were further characterized and chosen as a biocatalyst for the synthesis of IFOS.

### Effects of pH and temperature on the activity of free and immobilized inulosucrase

**Fig. 6:**
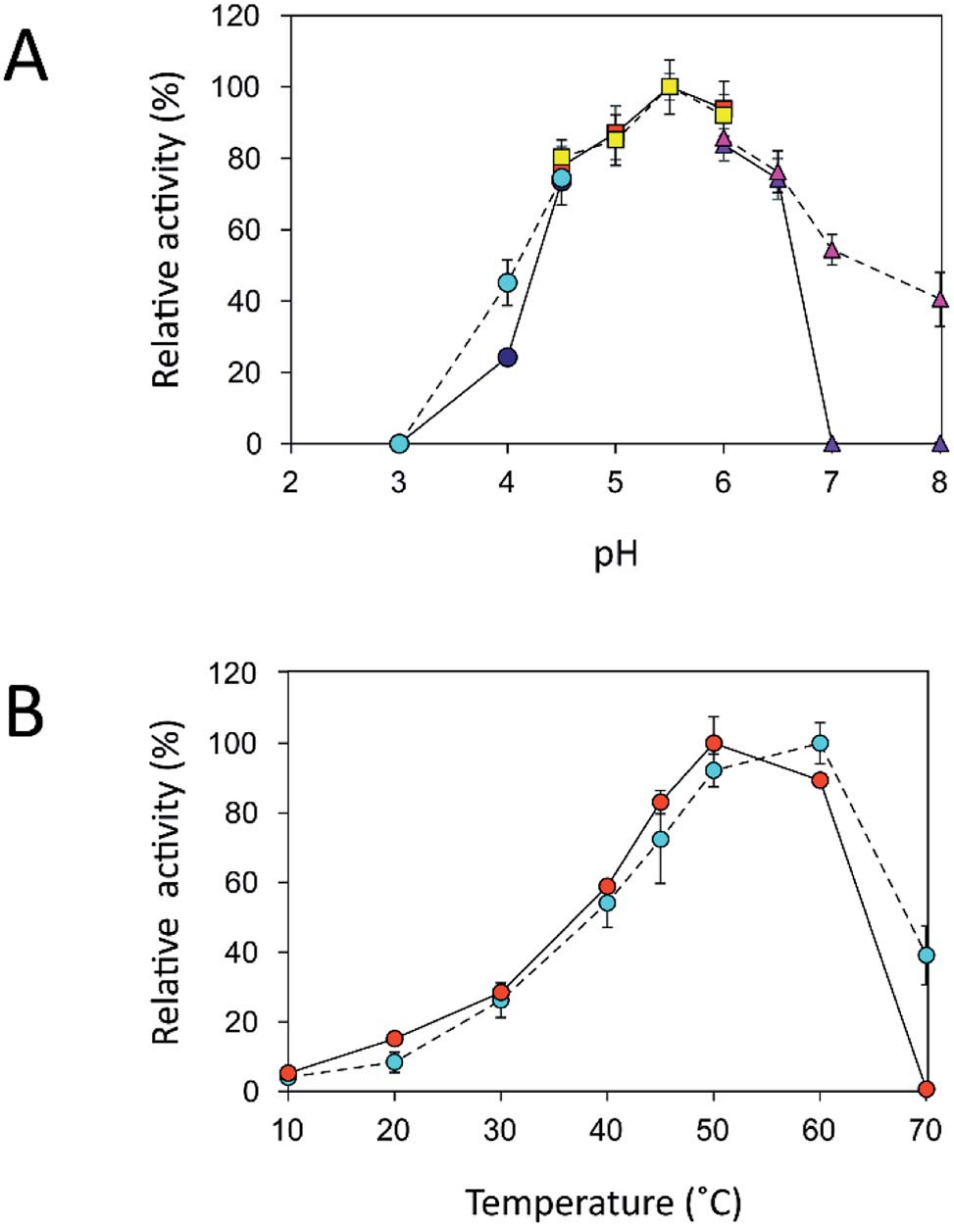
Biochemical characterization of free and immobilized inulosucrase. (A) Effects of pH on activity of free (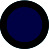, citrate buffer; 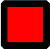, acetate buffer; 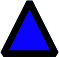, potassium phosphate buffer) and immobilized (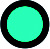, citrate buffer; 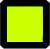, acetate buffer; 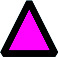, potassium phosphate buffer) inulosucrase when the reaction was carried out at 50 °C. (B) Effects of temperature on the activity of free (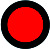) and immobilized (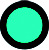) inulosucrase when the reaction was carried out at pH 5.5. Approximately 15 U of INU-CSBs or free inulosucrase were used for these experiments.

**Fig. 7:**
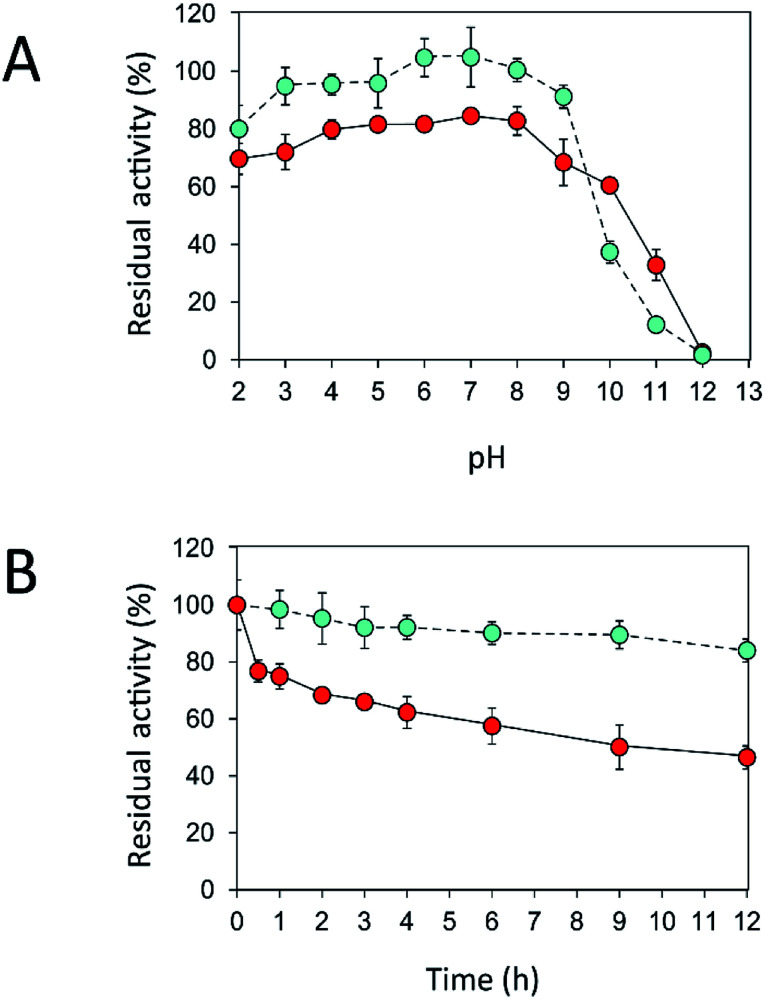
Effects of (A) pH and (B) temperature on stability of free (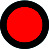) and immobilized (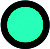) inulosucrase. Approximately 15 U of INU-CSBs or free inulosucrase were used for these experiments.

An immobilization process may change the kinetics and other properties of an enzyme.^36^ Enzymes are sensitive to changes in pH and can work best within their limited optimal pH range. After immobilization, the biochemical properties of INU-CSBs were studied and compared with those of the free enzyme. The free and immobilized enzymes had the same optimal pH, 5.5 (Fig. 6A), whereas the reaction temperature optima of inulosucrase were found to shift from 50 °C to 60 °C after immobilization (Fig. 6B). In addition, immobilization of inulosucrase showed a protective effect even at 70 °C, whereas the free enzyme lost all activity at the same temperature. This finding indicated that our immobilization technique gave greater stability to the enzyme, which was still able to function at a high temperature. The increase of optimal temperature of enzymes after immobilization had been reported in the case of *Aspergillus* β-glucosidase immobilized on chitosan.^37^ For analysis of pH stability, the retention of activity of both biocatalyst samples was measured after incubation at various pH levels at room temperature (30 °C) for 3 h. The results revealed that both free and immobilized enzymes were stable in a broad pH range: 2.0–10.0. Nonetheless, the enzyme of INU-CSBs was more stable than the free enzyme because it retained approximately 100% of its initial activity, whereas the free enzyme retained only 80% at pH 4.0–8.0 (Fig. 7A). The high stability of INU-CSB activity suggested that CSBs provided a microenvironment for enzyme molecules that prevented their conformational change during a pH change in the bulk solution. This phenomenon is known for immobilized α-amylase^38^ and tannase.^39^

Because activity and stability of inulosucrase are largely dependent on Ca^2+^ concentration,^40^ thermostability of the biocatalyst was studied in a buffer containing 40 mM CaCl_2_ (Fig. S4†). As shown in Fig. 7B, the thermostability of INU-CSBs was higher than that of the free enzyme. INU-CSBs retained ∼84% of activity after incubation at 50 °C for 12 h, whereas the residual activity of the free enzyme was lower than 50% under the same conditions. This phenomenon has been observed in many studies. For example, *Aspergillus aculeatus* β-fructofuranosidase that was immobilized on chitosan beads retains ∼100% of activity after incubation at 50 °C for 50 h, while the free β-fructofuranosidase retains only 50% of activity at the same temperature.^8^ β-Fructofuranosidase from *Aspergillus japonicus* loses almost all the activity after incubation at 37 °C for 7 days, whereas the immobilized enzyme retains approximately 60%.^7^ The enhanced thermostability of INU-CSB may result from the increase of enzyme rigidity after covalent linking onto the support. This approach has provided many advantages for industrial applications. For example, inulosucrase needs to use sucrose as a substrate, but at a high concentration of a sucrose, the solution is viscous. The reaction then needs to be conducted at a higher temperature to lower the viscosity. In addition, at the temperature higher than 50 °C, the growth of some pathogenic microorganisms will be stopped.^41^

### IFOS synthesis in batch mode on INU-CSBs

**Fig. 8:**
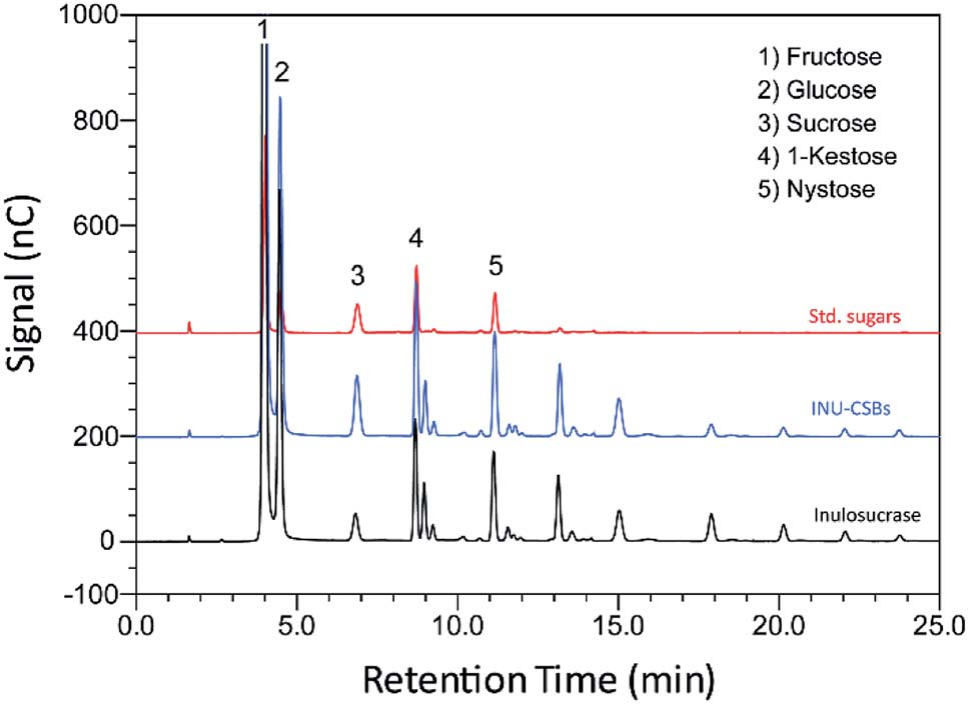
An HPAEC chromatogram of standard sugars, IFOS synthesized by INU-CSBs and free inulosucrase. IFOS was synthesized by incubating 10 U mL^−1^ biocatalysts with 200 g L^−1^ sucrose at 40 °C for 24 h.

Although immobilization of an enzyme through multiple covalent bonds provides a stable biocatalyst, it may cause a conformational change of the enzyme.^42^ Because 3D structure of an enzyme determines the specificity for substrates or products that they catalyze or synthesize, it is possible that the product patterns of a free and immobilized enzyme may be different. To investigate the effect of immobilization on the IFOS profile, a qualitative analysis of IFOS derived from both free and immobilized enzymes was performed by HPAEC-PAD. In this work, IFOS was synthesized by incubating 10 U mL^−1^ INU-CSB or free enzyme with 200 g L^−1^ sucrose at pH 5.5 and 40 °C for 24 h. HPAEC analysis showed that the pattern of products synthesized by INU-CSBs and free enzyme were comparable (Fig. 8). The IFOS products contained at least eight different oligosaccharides. In comparison with 1-kestose and nystose standards, the resulting oligosaccharides were IFOSs in which fructose is covalently linked *via* the β(2→1) linkage. The results indicated that immobilization did not affect the product pattern of the enzyme.

One of the advantages of the immobilized enzyme in industrial applications is its reusability. The operational stability of INU-CSB swas evaluated in a series of batch reactions. The IFOS synthesis by INU-CSB was performed at 200 g L^−1^ sucrose as a substrate. After 2 h of incubation of each batch, the retained activity of INU-CSBs was measured, and IFOS content was then analyzed.

**Fig. 9:**
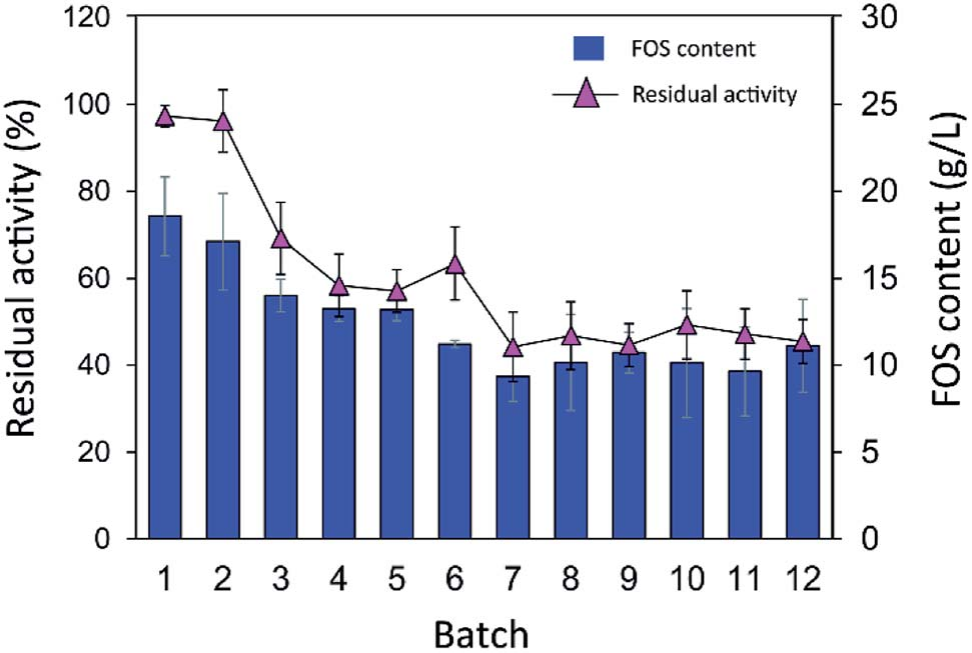
Batch reusability of INU-CSBs for IFOS synthesis. Reaction condition: 10 U mL^−1^ of biocatalysts were incubated with 200 g L^−1^ sucrose in acetate buffer pH 5.5, 40 °C and 2 h per batch.

As shown in Fig. 9, the IFOS amount synthesized by the first batch was the highest, 70.9 ± 8.7 g L^−1^, with the yield of 35.4% ± 4.4%. After that, it gradually decreased and seemed to be constant at ∼30 g L^−1^ IFOS (16% yield) after six cycles of reuse. The reduction of the IFOS amount in the early cycles correlated with the retained activity of the biocatalyst. INU-CSBs retained ∼60% of activity after the first 4 cycles and remained quite stable at approximately 45% even though it was reused for 12 cycles. This loss of activity in early cycles is characteristic of covalent immobilization because enzyme molecules that are noncovalently attached to the carrier may be desorbed by polarity of a sugar solution.^35^

Moreover, it is possible that the population of enzyme molecules that was differently attached to the support may be less stable than the others. These molecules may be denatured or degraded after early cycles of reuse, whereas some enzyme molecules still retain their activity even after many cycles of reuse. In comparison, in other studies, the operational stability of levansucrase immobilized on chitosan beads was studied at only 5 min per cycle; the immobilized enzyme lost ∼40% of its initial activity after working for 85 min (17 cycles).^43^ According to Santos-Moriano's report, after only 3 cycles of reuse of levansucrase immobilized on vinyl sulfone-activated silica, approximately 60% of activity was retained, and the reaction was allowed to proceed for only 20 min per cycle.^35^ In our study, each batch reaction time was 2 h. The immobilized enzyme was found to retain as much as 45% of its activity after 12 repeated uses for 24 h in total.

### IFOS synthesis in a continuous fixed-bed bioreactor on INU-CSBs

**Fig. 10:**
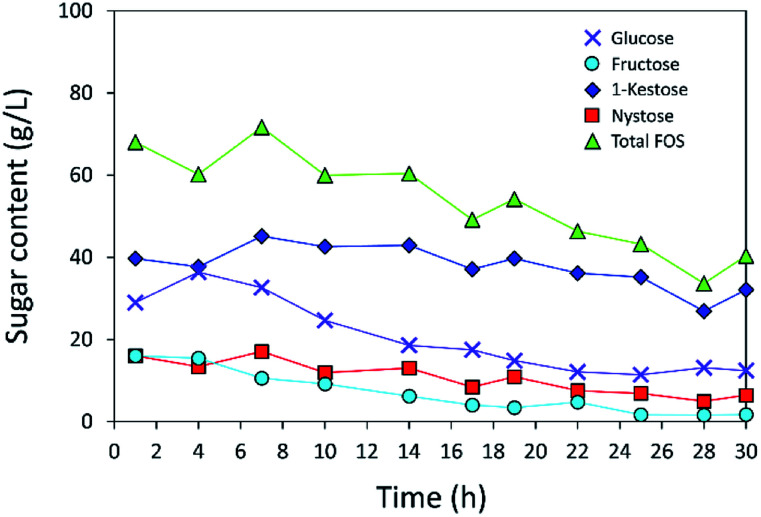
Continuous production of IFOS by immobilized inulosucrase in the fixed-bed reactor in which INU-CSBs (∼200 U in total) were packed into small double-jacket column. Sucrose at a concentration of 200 g L^−1^ was fed at a flow rate of 0.2 mL min^−1^. This process was conducted at 40 °C for 30 h.

The operational stability of the immobilized inulosucrase system was also investigated in a continuous fixed-bed reactor. A double-jacket column was packed with INU-CSBs. The continuous synthesis of IFOS was operated by feeding 200 g L^−1^ sucrose at a flow rate of 0.2 mL min^−1^ and 40 °C for 30 h. IFOS syrup was sampled and analyzed at certain intervals by HPLC. As shown in Fig. 10, the INU-CSB fixed-bed reactor synthesized various types of IFOSs, mainly 1-kestose, at least for 30 h with the average final total IFOS concentration of 53.0 g L^−1^. Although total IFOS content gradually decreased during initial operating time (14 h), it was nearly constant when the operating time was up to 30 h. Moreover, readers can clearly see that 1-kestose was constantly synthesized, with the average amount of approximately 37 g L^−1^. These results indicated that INU-CSBs have potential applications to the production of IFOSs from sucrose in both batch and continuous processes.

## Conclusions

This is the first study to show immobilization of a bacterial inulosucrase and the use of this immobilized enzyme in IFOS synthesis. It is evident that chitosan beads in core–shell format can overcome some limitations of traditional hydrogel beads and thus may be useful for the development immobilization methods for other enzymes. Inulosucrase immobilized on CSBs manifested resistance to thermal denaturation and has promising operational stability for batch and continuous production of IFOSs. These properties make INU-CSBs highly attractive as an alternative biocatalyst for the synthesis of IFOSs and for future biotechnological applications.

## Conflicts of interest

There are no conflicts to declare.

## Supplementary Material

RA-008-C8RA02241K-s001
