# Correction: Highly porous core–shell chitosan beads with superb immobilization efficiency for *Lactobacillus reuteri* 121 inulosucrase and production of inulin-type fructooligosaccharides

**DOI:** 10.1039/c9ra90009h

**Published:** 2019-02-05

**Authors:** Thanapon Charoenwongpaiboon, Karan Wangpaiboon, Rath Pichyangkura, Manchumas Hengsakul Prousoontorn

**Affiliations:** Department of Biochemistry, Faculty of Science, Chulalongkorn University Payathai Road Bangkok 10330 Thailand manchumas.h@chula.ac.th

## Abstract

Correction for ‘Highly porous core–shell chitosan beads with superb immobilization efficiency for *Lactobacillus reuteri* 121 inulosucrase and production of inulin-type fructooligosaccharides’ by Thanapon Charoenwongpaiboon *et al.*, *RSC Adv.*, 2018, **8**, 17008–17016.

The authors regret that Fig. 9 in the original article was displayed incorrectly. The correct version is shown below.

**Fig. 9:**
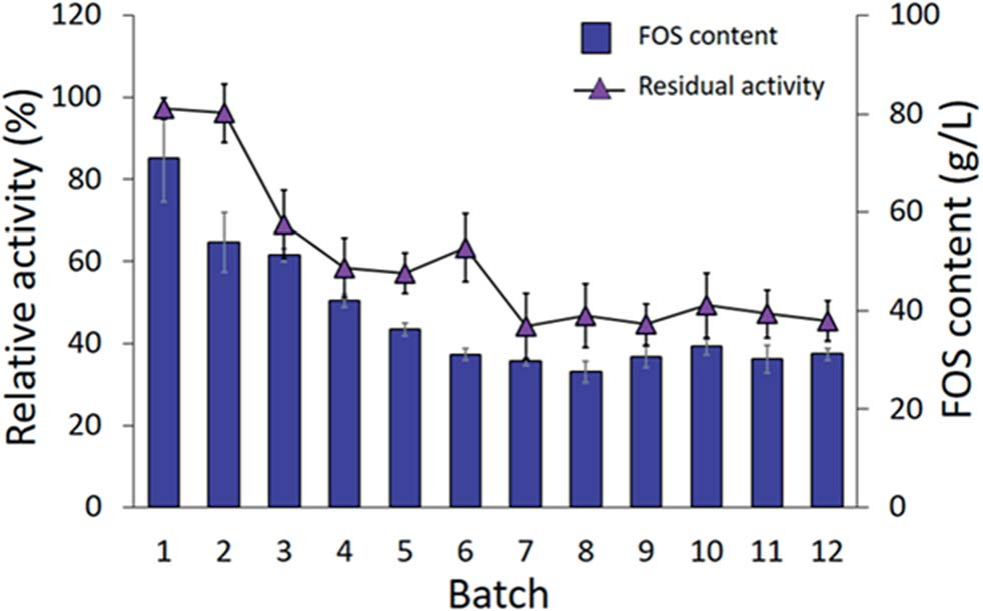
Batch reusability of INU-CSBs for IFOS synthesis. Reaction condition: 10 U mL^−1^ of biocatalysts were incubated with 200 g L^−1^ sucrose in acetate buffer pH 5.5, 40 °C and 2 h per batch.

The Royal Society of Chemistry apologises for these errors and any consequent inconvenience to authors and readers.

## Supplementary Material

