# Supplementary material for: Highly porous core–shell chitosan beads with superb immobilization efficiency for Lactobacillus reuteri 121 inulosucrase and production of inulin-type fructooligosaccharides
Source: RSC Adv. 2018 May 9;8(30):17008–16. doi: 10.1039/c8ra02241k (PMC9080446; doi:10.1039/c8ra02241k)
Supplement: RA-008-C8RA02241K-s001 [file RA-008-C8RA02241K-s001.pdf]

Electronic Supplementary Material (ESI) for RSC Advances.
This journal is © The Royal Society of Chemistry 2018

Supplementary Information

Highly porous core-shell chitosan beads with superb immobilization efficiency for *Lactobacillus reuteri* 121 inulosucrase and production of inulin-type fructooligosaccharides

Thanapon Charoenwongpaiboon, Karan Wangpaiboon, Rath Pichyangkura, Manchumas Hengsakul Prousoontorn*

Department of Biochemistry, Faculty of Science, Chulalongkorn University, Bangkok, Thailand

***Correspondence:** Dr. Manchumas hengsakul Prousoontorn, Department of Biochemistry, Faculty of Science, Chulalongkorn University, Phayathai Rd, Bangkok 10330, Thailand

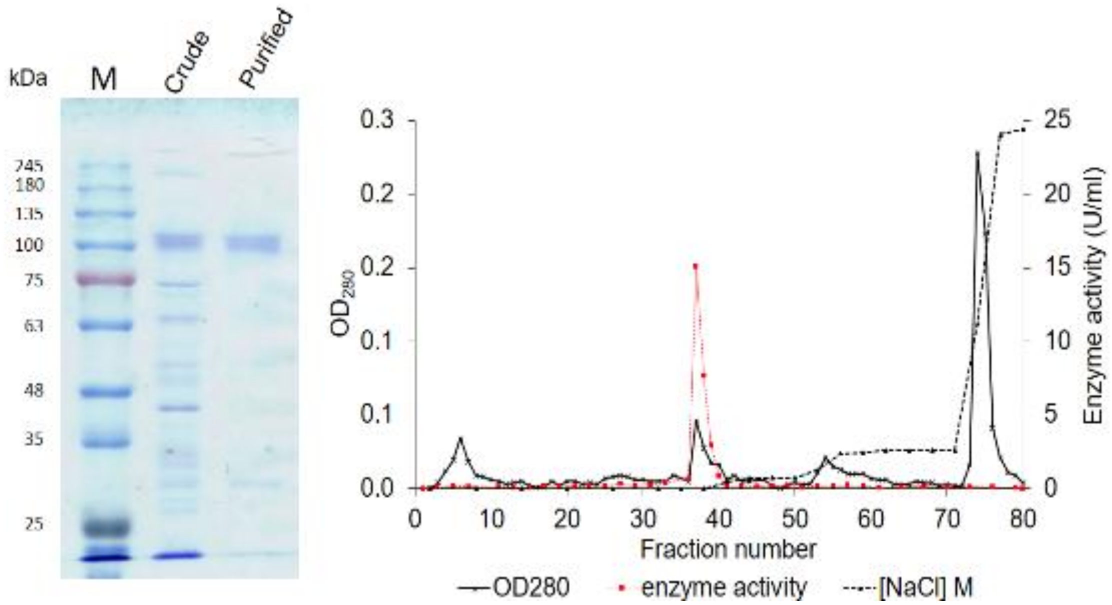

Fig. S1 Inulosucrase purification profile by DEAE toyopearl chromatography.

Table S1 Inulosucrase purification data.

step	Volume (ml)	Protein (mg)	Activity (U)	Specific activity (U/mg)	Purification (fold)	Yield (%)
Crude	20	18.3	1.23×10^3	67.0	1	100
DEAE-toyopearl	23	2.22	587	264	3.94	49

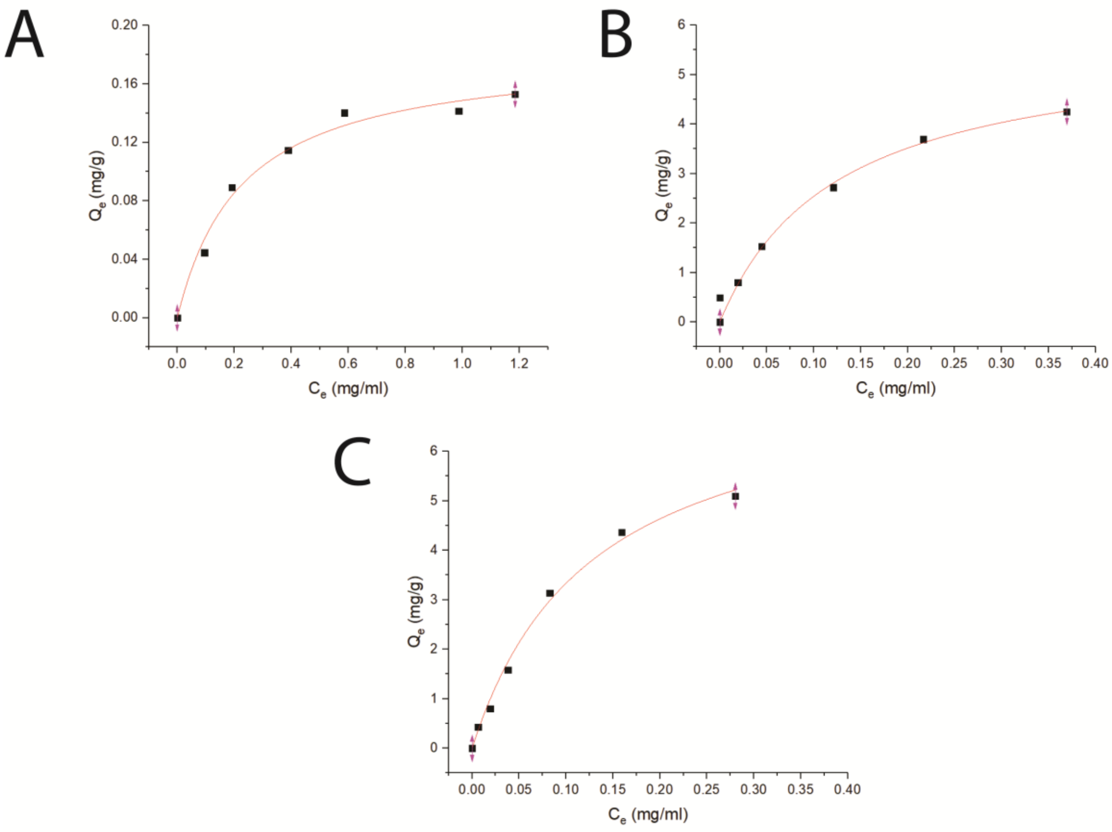

Fig. S2 BSA isotherm on DBs (A), HGBs (B) and CSBs (C) of the Langmuir model.

Table S2 Diameter of CSBs and DBs.

CSBs diameter (mm)				Shell/Core ratio	DBs diameter (mm)	
No.	Total	Core	Shell		No.	Total
1	1.19	1.08	0.11	0.10	1	1.43
2	1.30	1.21	0.09	0.07	2	1.23
3	1.27	1.14	0.13	0.11	3	1.30
4	1.43	1.31	0.13	0.10	4	1.17
5	1.12	1.01	0.11	0.11	5	1.35
6	1.34	1.21	0.13	0.11	6	1.25
7	1.16	1.09	0.07	0.06	7	0.01
8	1.15	1.06	0.09	0.09	8	1.12
9	1.36	1.26	0.10	0.08	9	1.38
10	1.35	1.25	0.10	0.08	10	1.32
11	1.19	1.12	0.07	0.06	11	1.23
12	1.17	1.07	0.11	0.10	12	1.42
13	1.24	1.14	0.10	0.09	13	1.21
14	1.31	1.19	0.12	0.10	14	1.37
15	1.29	1.18	0.11	0.09	15	1.19
16	1.40	1.30	0.10	0.08	16	1.50
17	1.20	1.09	0.12	0.11	17	1.35
18	1.15	1.10	0.05	0.05	18	1.20
19	1.29	1.20	0.09	0.08	19	1.24
20	1.17	1.10	0.08	0.07	20	1.07
21	1.26	1.17	0.08	0.07	21	1.21
22	1.27	1.17	0.10	0.08	22	1.36
23	1.11	1.04	0.07	0.07	23	1.41
24	1.21	1.13	0.09	0.08	24	0.97
25	1.12	1.02	0.10	0.10	25	1.33
26	1.47	1.34	0.13	0.10	26	1.39
27	1.30	1.18	0.12	0.10	27	1.40
28	1.40	1.27	0.13	0.10	28	1.28
29	1.07	0.99	0.08	0.08	29	1.39
30	1.32	1.22	0.11	0.09	30	1.28
31	1.14	1.05	0.09	0.08	31	1.17
32	1.54	1.35	0.19	0.14	32	1.17
33	1.19	1.09	0.10	0.09	33	1.34
34	1.36	1.20	0.16	0.14	34	1.30
35	1.24	1.08	0.16	0.15	35	1.35
Mean	1.26	1.15	0.11	0.09	Mean	1.25
SD	0.11	0.10	0.03	0.02	SD	0.24

A

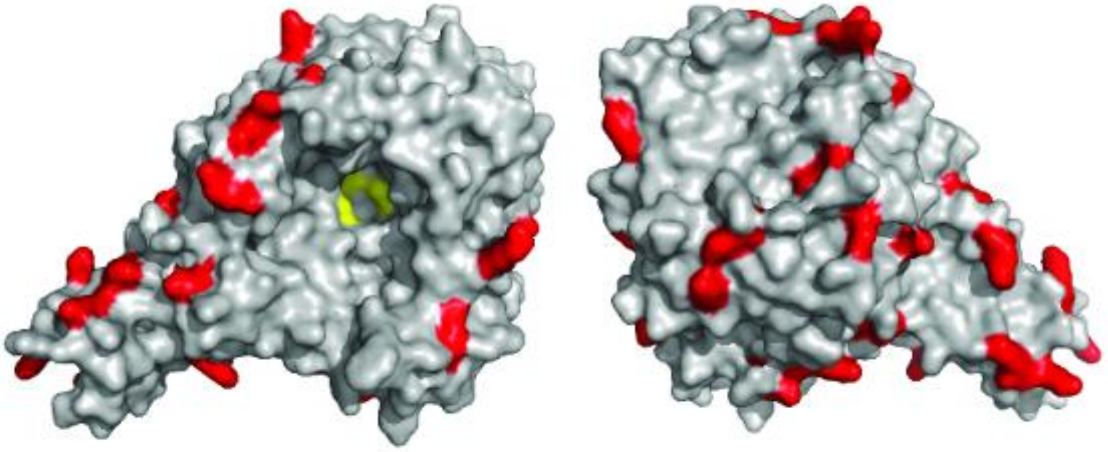

B

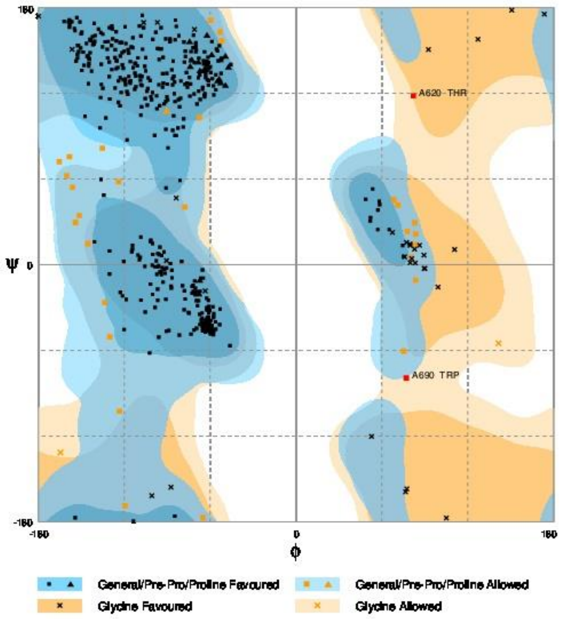

Fig. S3 (A) Homology modeling of inulosucrase of *Lactobacillus reuteri* 121 by means of Swissmodel [1-5], using the 3D structure of inulosucrase from *Lactobacillus johnsonii* NCC 533 (Protein Data Bank [PDB] ID: 2YFR, 73% identity) as a template. On the left is a frontal view, and on the right is the posterior view of protein; Lys residues are red, and three catalytic residues are yellow. (B) Ramachandran Plot for homology modeling of Inu calculated by RAMPAGE sever (http://mordred.bioc.cam.ac.uk/~rapper/rampage.php) [6].

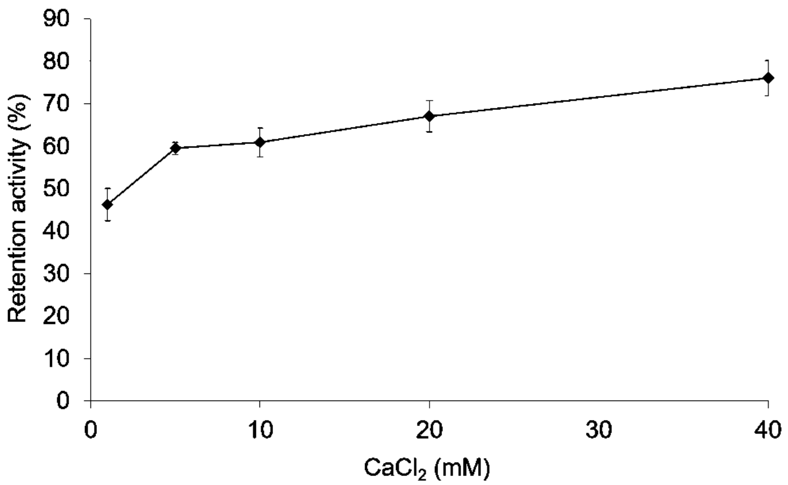

Fig. S4 Effect of $CaCl_2$ concentration on INU-CSBs stability.

Reference

1. Bienert, S., et al., *The SWISS-MODEL Repository—new features and functionality.* Nucleic Acids Research, 2017. **45**(Database issue): p. D313-D319.
2. Benkert, P., M. Biasini, and T. Schwede, *Toward the estimation of the absolute quality of individual protein structure models.* Bioinformatics, 2011. **27**(3): p. 343-350.
3. Guex, N., M.C. Peitsch, and T. Schwede, *Automated comparative protein structure modeling with SWISS-MODEL and Swiss-PdbViewer: A historical perspective.* ELECTROPHORESIS, 2009. **30**(S1): p. S162-S173.
4. Biasini, M., et al., *SWISS-MODEL: modelling protein tertiary and quaternary structure using evolutionary information.* Nucleic Acids Research, 2014. **42**(W1): p. W252-W258.
5. Bertoni, M., et al., *Modeling protein quaternary structure of homo- and hetero-oligomers beyond binary interactions by homology.* Scientific Reports, 2017. **7**: p. 10480.
6. Lovell, S.C., et al., *Structure validation by Cα geometry: ϕ,ψ and Cβ deviation.* Proteins: Structure, Function, and Bioinformatics, 2003. **50**(3): p. 437-450.
